# How Chemotherapy Increases the Risk of Systemic Candidiasis in Cancer Patients: Current Paradigm and Future Directions

**DOI:** 10.3390/pathogens5010006

**Published:** 2016-01-15

**Authors:** Flora Teoh, Norman Pavelka

**Affiliations:** 1 Singapore Immunology Network, Agency for Science, Technology and Research (A*STAR), 8A Biomedical Grove, Immunos Building, Singapore 138648, Singapore; flora_teoh@immunol.a-star.edu.sg; 2 School of Biological Sciences, Nanyang Technological University, 60 Nanyang Drive, Singapore 637551, Singapore

**Keywords:** chemotherapy, cancer, *Candida albicans*, immune system, microbiota

## Abstract

*Candida albicans* is a fungal commensal and a major colonizer of the human skin, as well as of the gastrointestinal and genitourinary tracts. It is also one of the leading causes of opportunistic microbial infections in cancer patients, often presenting in a life-threatening, systemic form. Increased susceptibility to such infections in cancer patients is attributed primarily to chemotherapy-induced depression of innate immune cells and weakened epithelial barriers, which are the body’s first-line defenses against fungal infections. Moreover, classical chemotherapeutic agents also have a detrimental effect on components of the adaptive immune system, which further play important roles in the antifungal response. In this review, we discuss the current paradigm regarding the mechanisms behind the increased risk of systemic candidiasis in cancer patients. We also highlight some recent findings, which suggest that chemotherapy may have more extensive effects beyond the human host, in particular towards *C. albicans* itself and the bacterial microbiota. The extent to which these additional effects contribute towards the development of candidiasis in chemotherapy-treated patients remains to be investigated.

## 1. Introduction

The World Health Organization has estimated 8.2 million cancer-related deaths worldwide, with 14 million new cases in 2012 and an increase of new cases by ~70% over the next 20 years [1]. Chemotherapy is one of the primary therapeutic interventions in cancer. Together with other medical interventions—such as radiotherapy and surgery—it has helped to increase the life expectancy of cancer patients and, in some cases, has led to complete remission [2,3,4].

However, classical chemotherapeutic agents are well-known to be non-specific, targeting rapidly-dividing cells with little discrimination between healthy and cancerous tissues. This lack of specificity leads to side effects ranging from cosmetic (e.g., hair loss), to relatively mild (such as fatigue, nausea and vomiting), and even potentially life-threatening consequences (immunosuppression and organ damage). Overall, these side effects reduce patients’ quality of life [5] and also, ironically, expose them to a greater risk of death from some other cause. One such cause is opportunistic microbial infections [6]. Common microbes which ordinarily pose no threat to healthy individuals can cause potentially lethal disease in the immunocompromised population [7], of which cancer patients make up a significant proportion [8]. In addition to interfering with immune cell development, chemotherapy affects the self-renewal of epithelial cells, thus causing disruptions of barriers that normally defend the host against invasion by microorganisms [9,10].

A leading cause of opportunistic microbial infections are fungal species belonging to the *Candida* genus, with *C. albicans* ranking as the most prevalent causative agent of candidemia around the world [11,12,13,14,15,16,17,18]. This trend has been observed over the past decade and is still the case, even in developed countries such as the United States, Denmark, Norway, and Finland [13]. *C. albicans* is a human commensal and a major resident of the skin, mucosal surfaces, the gastrointestinal (GI) tract and the female genitourinary tract [19]. It can cause superficial disease in otherwise healthy individuals, but infection in immunocompromised individuals can progress towards the potentially lethal systemic form. While infection arising from external sources (such as catheter colonization) can, and does, occur, evidence points to the patient’s natural colonizers as one of the primary sources of systemic candidiasis [20,21]. Most studies place the crude mortality rate due to *C. albicans* infections at around 30%–40% [11,15,16,22], but some have estimated this to be as high as 46%–75% [23]. Additional challenges associated with managing systemic candidiasis include the lag time between onset of symptoms and initiation of antifungal therapy due to difficulties in diagnosis, and an increasing rate of resistance against commonly used antifungal drugs [7,23]. Its impact on the healthcare system is considerable, as the disease is associated with longer hospital stays, and the average cost of illness has not decreased in over a decade. In 1998 direct medical costs were reported to range from ~$34,000 to $44,000 per patient in the United States [24], and in 2010, Moran *et al.* reported costs to range from $32,810 to $52,112 [25]. There is also the intriguing hypothesis that *C. albicans* infection itself can cause tumor progression and metastasis [26]. For these reasons, understanding the mechanisms underlying systemic candidiasis in susceptible patients will be important to develop novel strategies of prevention and treatment of the disease.

This review will cover the changes induced in the mammalian host by chemotherapy treatment, ranging from well-established effects, such as immunosuppression and epithelial barrier disruption, to more recently discovered effects on the host GI microbiota (including *C. albicans* itself), and how these may impact disease risk and outcomes in the context of systemic candidiasis.

## 2. Epidemiology of Systemic Candidiasis in Cancer Patients

*C. albicans* is one of the most common causes of bloodstream infections in cancer patients [27]. The average annual incidence of systemic candidiasis in cancer patients ranges from 71 to 2400 per 100,000 admissions, compared to 10–370 per 100,000 admissions in the general hospital population [28]. Neutropenia is commonly cited as an important risk factor for the development for systemic candidiasis [28,29]. It can be caused by certain types of blood cancers or cancers infiltrating the bone marrow, and is a common side effect of classical chemotherapy [30,31]. However, recent epidemiological data—focusing specifically on *C. albicans*—suggests that neutropenia may not be the sole predisposing factor for systemic infection in cancer patients. Several studies showed how neutropenic patients comprised only 10% or less of all patients with systemic *C. albicans* infection [15,16,17]. A review by Nesher also finds that the risk of neutropenia-related infections was greater in patients with hematological malignancies than those with solid tumors [32], yet patients with solid tumors are far more likely to develop systemic *C. albicans* infection than those with blood cancers [11,15,16,17]. This difference may be due to the specific chemotherapeutic agents used to treat different types of cancer, which may have varying effects on the host immune system (see below); or they may be due to differences in the use of various medical interventions, such as catheter use or surgical procedures, which are, themselves, risk factors for the development of systemic candidiasis [28]. Other risk factors for fungal infections, which are also commonly found in cancer patients, are mechanical ventilation [33], corticosteroid therapy, and renal failure [34,35].

## 3. Effects of Chemotherapy on the Immune System

### 3.1. Effects on Innate Immunity

#### 3.1.1. Cellular Factors

Neutrophils play a key role in the host response against *C. albicans*. They are among the first immune cells to be recruited at the site of infection via pro-inflammatory cytokines secreted by epithelial cells and macrophages, and respond by releasing antimicrobial peptides (AMPs) and reactive oxygen species (ROS) [36]. Moreover, neutrophils phagocytose the yeast form of *C. albicans* [37] and form neutrophil extracellular traps (NETs), which assist in extracellular killing of hyphae that are often too large to be phagocytosed [38,39] or even contribute to the ability of the fungi to escape from phagocytes [40,41]. The essential role of neutrophils in antifungal defense is illustrated by the fact that mice treated with neutrophil-depleting antibodies exhibited more extensive tissue damage in several organs alongside a greater fungal burden in the kidney (a common site of invasion in the murine model for acute disseminated candidiasis) upon systemic challenge with *C. albicans* [42]. However, it has been shown that neutrophil depletion on its own cannot induce dissemination of *C. albicans* from the GI tract, and systemic infection only occurs when neutropenia is accompanied by GI barrier disruption [43].

While having sufficient numbers of neutrophils is necessary for effective host defense, normal neutrophil function is also an important consideration, as evidenced by the increased risk of bacterial and fungal infection in patients with a normal neutrophil count but poor neutrophil function [44,45]. In addition to reducing the absolute number of neutrophils, chemotherapy treatment can also induce decreased migratory and phagocytic activity in these cells, which has been correlated with poorer candidacidal activity [46]. Specific chemotherapeutic agents that perturb microtubule components (such as α- and β-tubulin) may also interfere with phagocytic and chemotactic activity, as these are all dynamic cytoskeletal processes [47,48]. Microtubule poisons (such as paclitaxel, docetaxel, and vincristine) which act on cancer cells by disrupting the mitotic spindle may, thus, also impair fungal clearance by interfering with normal microtubule dynamics for phagocytosis and migration [49]. One study has shown that neutrophils from breast cancer patients treated with anthracyclines exhibit reduced actin polymerization in neutrophils upon stimulation with IL-8, compared to before the start of treatment, possibly suggesting an impairment in the normal cytoskeletal dynamics [50]. Exactly how anthracyclines may perturb actin dynamics is not clear, as their main mode of action is the inhibition of topoisomerases.

Chemotherapy treatment also appears to result in the increased release of DNA-histone complexes from neutrophils [51]. These complexes were found to possess pro-coagulant activity and suggested to be a contributing factor in the increased rate of thrombosis in chemotherapy-treated breast cancer patients [51]. Since NETs, which are formed by DNA-histone complexes, have also been shown to facilitate platelet adhesion and aggregation [52], together these results suggest that chemotherapy might stimulate NETosis. One would expect an increased amount of NETs to facilitate extracellular killing of pathogens; however, it needs to be evaluated whether cytotoxic chemotherapy agents that induce chemical alterations in DNA strands (e.g., alkylating agents) may also change the functionality of NETs. Consistent with this possibility, it has been observed that extracellular DNA demonstrates antimicrobial properties that can be abrogated by DNase I, alkaline phosphatase, and excess Mg^2+^ [53]. Extracellular DNA generated by NETosis is known to have similar antimicrobial properties, which could potentially be affected by such chemical treatments.

Monocytes and macrophages also play an important role in anti-*Candida* defense, as their specific depletion results in mice that more rapidly succumb to systemic *C. albicans* infection [54,55]. Like neutrophils, they are capable of phagocytosing and killing *C. albicans* [37], in addition to secreting pro-inflammatory cytokines that contribute to the antifungal response [36]. They have also been shown to confer a memory-like protection against a systemic challenge of *C. albicans* after priming with a sublethal dose of the fungus [56]. This phenomenon is now known as “trained immunity”, and has been shown to be due to a combination of epigenetic and metabolic reprogramming of monocytes and macrophages [57,58]. In particular, the β-glucan-induced differentiation of naïve monocytes into “trained” macrophages involves epigenetic remodelling in cell signaling modules such as the cAMP pathway, which was shown to be crucial in mediating protection against subsequent *C. albicans* infection [58]. In parallel, a metabolic switch from oxidative phosphorylation to aerobic glycolysis was induced in monocytes upon Dectin-1-mediated recognition of *C. albicans* β-glucan, and was also crucial for the protective effects of trained immunity [57]. Hence, monocytes and macrophages play a fundamental role in protecting the host from *C. albicans* infections and re-infection and any perturbation of their number and function by chemotherapy is therefore likely to increase the risk of candidiasis.

Monocytopenia has been observed early after cisplatin-based treatment in humans [59]. Functional changes in monocytes and macrophages have also been reported after chemotherapy. Cyclophosphamide treatment was found to lead to the down-regulation of both CX3CR1 and its ligand CX3CL1 on immature monocytes, promoting their efflux from the bone marrow and increasing their availability in the circulation [60]. However, another study showed that monocyte-specific CX3CR1 deficiency results in increased fungal burden in the kidney and a poorer survival upon systemic *C. albicans* infection in mice [61]. This was associated with a reduced number of kidney monocytes as a result of increased apoptosis. The mechanism behind how CX3CR1 improves survival and persistence in kidney monocytes is not clear. CX3CR1-deficient monocytes did not, surprisingly, demonstrate defective trafficking from blood to kidney [61], despite this molecule’s prominent role as a chemokine receptor. Overall, these findings demonstrate how chemotherapeutic treatment may perturb both the levels and function of monocytes and macrophages during systemic *C. albicans* infection in a manner that may have a detrimental effect on host antifungal defense.

NK cells represent another important component of the innate immune system with well-known roles in antitumor responses and viral infections, but their contribution to antifungal defense is less well understood. Recent studies showed that human NK cells directly recognize *C. albicans* [62] and that these immune cells are crucial in the control of systemic *C. albicans* infection especially in mice lacking T and B cells [63]. This was dependent on the developmental programming of NK cells induced by IL-17 signaling [64]. Mice deficient in IL-17RA showed reduced generation of GM-CSF-producing NK cells, and these cells were required to mediate effective fungal killing by neutrophils. Neutrophils in IL-17RA-deficient mice did not display an intrinsic defect in their fungicidal ability, but required these GM-CSF-producing NK cells for activation and survival [64]. Interestingly, a reduction in the number and function of NK cells following chemotherapy was found in melanoma patients [65], although the impact of these chemotherapy-induced side effects in the context of systemic *C. albicans* infection has yet to be investigated.

#### 3.1.2. Humoral Factors

In addition to cellular components, humoral factors such as AMPs also play a prominent role in innate immunity. Some AMPs are known to have inhibitory effects against *C. albicans* (reviewed by Swidergall and Ernst [66]). There is little existing literature on how chemotherapeutic agents affect the production of AMPs. One study shows that tegafur (a pro-drug metabolized by the liver to 5-fluorouracil) caused a reduction in the mRNA expression of α-defensin-5 and -6 in Caco2 cells, at least in the early stages of treatment. This reduced expression was also correlated with increased production of ROS, although it remains unclear if there was a causative association between these two observations. Both α-defensin-5 and 6 are expressed by Paneth cells in the gut [67,68], a well-known reservoir of *C. albicans* in the human host. Both of these defensins are known to be active against bacteria, and α-defensin-5 is known to be active against *C. albicans* as well [69]. However the study did not test the expression of other defensins that might also be affected by chemotherapeutic treatment, such as α-defensin-1, which is better known for its activity against *C. albicans* [70].

β-defensins-2 and -3 are other AMPs with fungicidal activity against *C. albicans*, although their modes of action appear to differ [71,72]. How they may be affected by chemotherapeutic treatment remains to be studied. Meyer *et al.* examined how pre-incubation of oral epithelial cells with the corticosteroid betamethasone resulted in reduced β-defensin-2 expression, upon stimulation with heat-killed *C. albicans* [73]. Corticosteroids are commonly prescribed to ameliorate common side effects arising from cytotoxic chemotherapy such as nausea, vomiting [74], and hypersensitivity reactions [75] and, thus, may be indirectly implicated in the reduced host defense against *C. albicans* infection arising from chemotherapy treatment.

### 3.2. Effects on Adaptive Immunity

#### 3.2.1. Cellular Factors

In addition to innate immune cells, chemotherapy also causes a general reduction in cells of the adaptive immune system [76,77]. Although deficiencies in adaptive immunity lead to an increased susceptibility to mucocutaneous candidiasis but not systemic infection [78], careful orchestration of the adaptive immune response is still important in protection against *C. albicans*. Indeed, different T cell subsets play intricate roles in organizing the antifungal host response by effector cells like neutrophils. This has been reviewed extensively by Romani [79], and we briefly summarize the main points as follows: during fungal infection T_H_1 cells facilitate pathogen clearance due to their enhancement of phagocyte antimicrobial activity via interferon-γ secretion [80,81], and their involvement is associated with a protective response during systemic infection [82,83]. T_H_17 cells secrete IL-17 that serves as a chemoattractant for neutrophils [84,85] and are essential for defense against epithelial invasion by *C. albicans* [86]. *Candida*-specific memory T cells also belong predominantly to this subset likely as a result of constant exposure to the fungus [87]. T_H_22 cells produce IL-22 and TNF-α which act synergistically to maintain epithelial layer integrity [88]. T_H_17 and T_H_22 cells are therefore primarily involved in mucosal defense. T_REG_ cells keep inflammation at the site of infection in check to reduce collateral damage to the host [89]; in healthy individuals, T_REG_ cells play an additional part in maintaining the commensal relationship with fungi and in protecting the host against fungal allergy [90]. Achieving a balance between these different responses is crucial for conferring both protection and tolerance to *C. albicans* [79]. Any perturbation to this balance by chemotherapy may thus hinder effective antifungal response by the adaptive immune system.

Lymphocyte depletion has been observed post-chemotherapy treatment since a long time [76,77]. Mackall *et al.* studied a group of ten patients, each with different cancers and varying chemotherapy regimens and found that despite the general reduction in levels of all blood cells seen in the patients, it was the lymphocyte population that took the longest amount of time to recover compared to neutrophils and monocytes [76]. The functional profiles of T lymphocytes were also altered, exhibiting increased HLA-DR expression (suggesting greater T cell activation), along with greater numbers of memory and effector T cells [76]. This observation has been suggested to be a possible indication of functional deficits in the T cell population [91]. To what extent these unequal effects of chemotherapy on different immune cell subsets can lead to unbalanced and, thus, pathogenic or ineffective, antifungal host responses remains to be investigated.

Among several chemotherapy drugs, cyclophosphamide has received particular attention for its ability to modulate antitumor immune responses as well as responses against *C. albicans*. Cyclophosphamide treatment preceding antitumor vaccination uncovers CD8^+^ T cells that take part in the antitumor response, as a result of T_REG_ suppression [92]. Other studies showed that cyclophosphamide administration in mice can improve or weaken resistance to systemic *C. albicans* challenge, depending on the amount of time by which cyclophosphamide administration precedes *C. albicans* challenge [93]. Interestingly, the improvement in antifungal response induced by cyclophosphamide was attributed to the appearance of cells from a monocyte/macrophage lineage.

On the other hand, a recent study by Litterman *et al.* has shown that treatment with alkylating chemotherapeutic agents dampened the antitumor adaptive immune response. Temozolomide treatment decreased T cell proliferation, which occurred in a dose-dependent manner and correlated with the DNA damage response in T lymphocytes. This response also correlated with a stronger TCR signaling, resulting in selection of T lymphocytes with poorer function. Similar observations were also made in cyclophosphamide-treated mice, but not those treated with doxorubicin or carboplatin [94], suggesting that the effect on the lymphocyte population is dependent on the type of DNA damage inflicted. While this study focused on antitumor vaccinations, it may be possible that such effects might also impact the intrinsic host antifungal defenses and the use of anti-*Candida* vaccines [95,96,97]. Indeed, anti-*Candida* vaccines are currently being considered as a means of preventing the development of candidiasis in susceptible populations [98]. Two of these vaccines—one derived from the agglutinin-like sequence 3 protein (Als3p0), and another derived from the secreted aspartyl protease 2 (Sap2)—have completed Phase I clinical trials and shown promising results [99,100]. Given that the protective effect of the Als3p vaccine was found to be mediated by T_H_1 and T_H_17 lymphocytes [101], it remains to be seen if this vaccine will demonstrate similar efficacy in individuals with chemotherapy-induced immunosuppression.

#### 3.2.2. Humoral Factors

In addition to cellular immune response, immunoglobulins have also been suggested to play an important role in the prevention and clearance of systemic candidiasis [102,103]. Chemotherapy treatment has a well-documented effect of reducing the levels of circulating antibodies. A proportion of pediatric patients diagnosed with blood cancers that had previously completed a national vaccination schedule (inclusive of measles, mumps, and rubella), displayed a reduction in antibody titers to non-protective levels, even one year after treatment was successful and completed [104,105]. Borella *et al.* observed that long-term combination chemotherapy also reduced serum IgG and IgA levels in response to influenza vaccination, possibly due to reduced numbers of B cells, although total IgM levels remained normal when compared to healthy untreated controls [106]. Moreover, Nilsson *et al.* observed a reduced number of bone marrow plasma cells (a subset of B cells) in their cohort of leukemia patients after chemotherapy treatment commenced, although the level of this cell population was already significantly depressed at the time of cancer diagnosis, and was further decreased with chemotherapy [104]. However, these studies were performed with primarily obligate pathogens in mind (influenza, mumps, Hepatitis B, rubella, *etc.*), and how well these results may be translated to respond to *C. albicans*, typically a human commensal persisting from birth, is not clear.

## 4. Effect of Chemotherapy on the Gut Epithelial Barrier and Resident Microbiota

### 4.1. Epithelial Barrier

Chemotherapy is well known for its detrimental effect on epithelial layers [107,108]. This effect of chemotherapy has, conventionally, been attributed to cell death of epithelial progenitors due to the non-specific effects of chemotherapy on rapidly-dividing cells [109,110]. This observation extends to the gut epithelium, as evidenced by the increased apoptosis in intestinal crypts observed post-chemotherapy treatment, followed by hypoplasia and atrophy [111]. Such disruption of the gut epithelium may facilitate invasion by pathogenic microbes such as *C. albicans* and their dissemination through the bloodstream [43].

There is a growing amount of evidence, however, that suggests that the increased gut permeability also results from chemotherapy-induced alterations in the tight junctions of the gut epithelium (as reviewed by Wardill and Bowen [112]), which are important for maintaining barrier integrity. Methotrexate (MTX) was found to reduce the expression of the tight junction proteins occludin and claudin in rats four days post-treatment [113]. *In vitro* experiments with Caco-2 monolayers also did not show a greater number of apoptotic cells upon MTX treatment, as one might expect from a conventional chemotherapeutic drug. Pre-treatment of monolayers with inhibitors of the MEK1, MEK2, NF-kB, and JNK pathways appeared to improve barrier integrity in epithelial cell monolayers after MTX treatment, implicating these pathways in MTX-induced gut epithelial barrier disruption [113]. However, the lower barrier integrity and tight junction protein expression were more pronounced in experiments using monolayers as opposed to *in vivo* experiments in rats [113]. Another study using MTX found that zonula-occludens-1 (another tight junction protein) expression is not reduced in MTX-treated rats, but its tyrosine phosphorylation is significantly decreased [114]. What this decrease in phosphorylation signified was not examined. It would be interesting to see how other chemotherapeutic drugs affect tight junction expression in the gut epithelium, whether there are any commonalities or differences depending on their modes of action, and also whether any significant microbial translocation actually takes place as a result of reduced barrier integrity.

### 4.2. Gut Microbiota

The gut-resident microbiota has been implicated in a diverse range of functions, from metabolism [115] and immune regulation [116], to the maintenance of epithelial barrier integrity [117]. In the case of *C. albicans*, which is also a co-resident in the human GI tract, the gut microbiota also plays a role in controlling *C. albicans* colonization and infection [118]. Specifically, certain metabolites produced by the gut microbiota, such as short-chain fatty acids, inhibit the yeast-hyphal transition in *C. albicans* [119] and restrict its growth [120]. Certain components of the gut microbiome have also been shown to mediate protective antifungal responses [86,121,122,123]. Consistent with the microbiota’s protective effects, antibiotic use is associated with *C. albicans* overgrowth [124] and is an independent risk factor for candidiasis [28,29,125].

Studies on the effect of chemotherapy on the microbiota are scarce, but initial findings in humans and animal models suggest it leads to reduced total number of bacteria in the gut [126,127], alongside alterations in gut microbiota composition [128]. However, these studies do not concur on exactly which bacterial populations are most significantly affected. Zwielehner *et al.* observed greater alterations in *Clostridium* cluster XIVa [127], while van Vliet *et al.* primarily reported marked differences in bifidobacteria and *Bacteroides* species [129]. These studies are limited by sample size, number of tested drugs, heterogeneity in pre-existing medical conditions within the study populations and variations in experimental methods, which may explain the inconsistent results. More systematic and comprehensive approaches would be needed to better understand how chemotherapy may alter the host gut microbiota and how these alterations might contribute to the increased risk of systemic candidiasis in cancer patients. Nevertheless, these findings demonstrate that chemotherapy is capable of perturbing the host gut microbiome and may, thus, directly or indirectly, influence the ability of the host to control *C. albicans* colonization and infection. However, chemotherapy-induced changes in the microbiota can sometimes be beneficial for the host, as shown from the discovery by Viaud *et al.* of a synergistic interaction between the gut microbiota and cyclophosphamide in boosting the host antitumor response [130].

## 5. Effect of Chemotherapy on *Candida Albicans*

As fungi are eukaryotes like mammalian cells, it is possible that chemotherapy will have similar effects on both. Indeed, hydroxyurea (used in treating ovarian cancer, leukemia, and melanoma [131]) is also a workhorse for basic cell cycle research in model organisms, such as the budding yeast *Saccharomyces cerevisiae*. This is one indication of the highly conserved nature of chemotherapy targets across eukaryotes. Since *C. albicans* colonizes the GI tract of most healthy individuals [19,132], it might thus have ample opportunity to get exposed to chemotherapeutic drugs in cancer patients undergoing treatment, and to be likewise affected by them. Several studies have already found hydroxyurea and a few other chemotherapeutic drugs to be capable of inducing morphological and phenotypic changes in *C. albicans* which, in turn, could potentially impact its virulence in the human host. Some of the traits commonly considered virulence factors in *C. albicans* (reviewed by Calderone *et al.* [133] and Mayer *et al.* [134]) are the ability to switch between different morphological forms, the ability to adhere to mammalian tissues, the production of certain proteinases as well as resistance to antifungal agents. It has been shown that some chemotherapeutic agents, namely cisplatin, 5-fluorouracil, and peplomycin all reduce the susceptibility of *C. albicans* to the antifungal drugs amphotericin B and miconazole, as well as improve adherence to HeLa cells [135]. Furthermore, hydroxyurea was found to induce filamentation in *C. albicans* [136], in addition to inducing phenotypic switching [137], a process primarily required for fungal mating [138] but also playing a role in biofilm formation [139], metabolic adaptation to different host microenvironments [140], and evasion from immune cells [141]. Uncovering chemotherapeutic drugs capable of affecting gut-resident *C. albicans* may yield clinically important findings about a potential contributing pathway to the development of systemic candidiasis, which has yet to be examined in detail.

## 6. Conclusions

**Figure 1:**
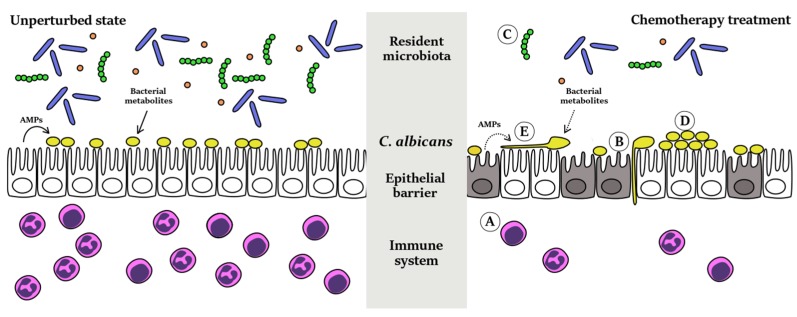
Pleiotropic effects of chemotherapy on the host, the microbiota, and *C. albicans*. The figure illustrates the physiological state in an unperturbed host asymptomatically colonized by *C. albicans*, and the changes that occur upon chemotherapy treatment. The grey column in the middle indicates the several points of interaction that chemotherapy makes with the host, the microbiota and the pathogen itself. Specific changes occurring during chemotherapy treatment are indicated by letters. A: The immunosuppressive effects of chemotherapeutic drugs result in the reduction in numbers and function of effector cells such as neutrophils, monocytes, macrophages, and lymphocytes, therefore weakening host defense against *C. albicans*. B: Maintenance and renewal of epithelial barriers is impeded by the action of chemotherapeutic agents on rapidly dividing cells. This leads to epithelial barrier disruptions that facilitate invasion by *C. albicans*. C: Chemotherapy may also lead to an overall reduction in microbiota abundance and/or an alteration of its composition. This may result in reduced production of bacterial metabolites that normally control colonization and virulence of *C. albicans*. D: Following chemotherapy, *C. albicans* overgrowth may occur as a result of (i) reduced production of antimicrobial peptides (AMPs) by epithelial cells and/or (ii) reduced level of bacterial metabolites that normally inhibit *C. albicans* growth. E: Chemotherapeutic agents might also induce hyphal formation in *C. albicans*, which would then facilitate invasion and entry into the bloodstream. Potential mechanisms include (i) the reduced production of certain bacterial metabolites that normally inhibit hyphal morphogenesis and/or (ii) direct effects of chemotherapy on *C. albicans* itself.

The central role that classical chemotherapeutic agents have played in the war on cancer cannot be denied. However, their non-specific nature makes them a double-edged sword, resulting in potentially life-threatening side effects, such as an increased risk of opportunistic microbial infections, of which *C. albicans* is a leading cause. Systemic candidiasis is highly prevalent in cancer patients and is associated with high morbidity, mortality and healthcare costs. Hence, in order to improve the overall survival and quality of life of cancer patients, we also need to consider ways to protect patients from such infections, alongside treating the disease itself. It is, therefore, necessary that we better understand how chemotherapeutic agents mediate the risk of opportunistic infection in order to find possible routes for disease interception. The disruption of epithelial barriers and alteration of host immune responses are well-established effects of chemotherapy that are known to increase susceptibility to systemic infection; furthermore, findings from recent studies suggest that chemotherapy may have wider-ranging effects on both *C. albicans* itself and on the commensal microbiota residing in the human host (schematically summarized in Figure 1). These unexpected effects could also directly contribute to, or indirectly modulate, the risk of opportunistic fungal infections in cancer patients and represent new and intriguing avenues for future studies and possible future interventions.
